# Clarithromycin-Induced Psychosis: When Is Delirium Not Delirium?

**DOI:** 10.7759/cureus.93096

**Published:** 2025-09-24

**Authors:** Scott R Coutts, Imogen Smith, Alexandra G Stirzaker

**Affiliations:** 1 Department of Medicine of the Elderly, St John's Hospital, National Health Service (NHS) Lothian, Livingston, GBR

**Keywords:** clarithromycin, delirium, macrolides, neuropsychiatric, psychosis

## Abstract

Clarithromycin-induced psychosis is a rare phenomenon; however, over the previous two decades, there has been increasing evidence linking psychotic episodes to the use of clarithromycin. The exact aetiology of this remains unclear, but several key hypotheses have arisen, including gamma-aminobutyric acid type A receptor (GABA-A) antagonism and active clarithromycin metabolites altering brain neurotransmitters such as glutamate. Recent evidence describes a broad range of symptoms, including delusions and catatonic states. Prior psychiatric comorbidities are a substantial risk factor. This case report describes the development of clarithromycin-induced psychosis in an 86-year-old patient with no prior psychiatric medical history, cognitive or functional impairment, and no other pathology found accountable for this neuropsychiatric disturbance. By acknowledging this rare adverse effect of clarithromycin and discontinuing the drug, the patient’s psychotic symptoms resolved within 24 hours. Given the widespread use of clarithromycin, this case report demonstrates the importance of being aware of its rare neuropsychiatric adverse effects to diagnose this relatively unusual presentation.

## Introduction

The British National Formulary (BNF) [1] reports psychotic disorders as an adverse reaction to clarithromycin (with frequency not known). Although hard to quantify, one study [2] demonstrated an incidence rate of 0.12 psychotic events per 1,000 clarithromycin prescriptions for a *Helicobacter pylori* eradication therapy. Several studies [2-4] document various neuropsychiatric disturbances with antibiotic use in adults, in particular macrolides. Of the macrolides, clarithromycin has been extensively researched, with studies citing clarithromycin-induced delusions and mania [5]. Evidence indicates psychotic symptoms associated with clarithromycin are often part of a delirium syndrome presenting acutely with a fluctuating course and impaired consciousness [4,6]. Delusions associated with clarithromycin are often intermittent, peculiar, and linked with the patient's environment, in contrast to structured theme-driven delusional thinking, which evolves gradually in primary psychotic disorders [4,6]. It is imperative to report adverse reactions, especially when the frequency remains unknown. There should be a degree of caution when prescribing clarithromycin in those with pre-existing mental health conditions or the frail elderly [2]. Awareness of this adverse effect is important to ensure prompt diagnosis when atypical features (such as psychosis) are prominent in patients with an acute confusional state [5].

This case report was previously presented as a poster at the Royal College of Psychiatrists in Scotland's Joint Old Age and General Adult Faculty Conference 2024 on December 6, 2024.

## Case presentation

An 86-year-old female sustained a thorn injury three days prior to admission and presented to her general practitioner (GP) with a suspected cellulitis overlying her thumb and fifth proximal phalanx. She was commenced on clarithromycin 500 mg twice daily by her GP. Three days later, she re-presented to her GP with acute confusion and was referred to the emergency department for further investigation. Her systemic enquiry was unremarkable, and her admission rapid 4 ‘A’s test (4AT) (reproduced with permission from www.the4AT.com under the Creative Commons "BY" license) score was 8 [7]. On examination, she was systemically well with no fever. She had mild swelling on the dorsum of her hand, accompanied by erythema, but no evidence of a deep infection, such as a flexor sheath infection (e.g., no tenderness on passive extension). She was oriented to place and able to follow one-stage commands. She exhibited echolalia (often repeating instructions given to her).

She had a past medical history of ventricular desynchrony (dual chamber cardiac pacemaker in situ), ischaemic heart disease, hypertension, arthritis, and aortic stenosis with severe left ventricular outflow obstruction. Prior to admission, she was functionally independent, with a Clinical Frailty Score of 2 (used with permission for research and educational purposes from the original copyright holders), and had no psychiatric history or concerns regarding cognition [8]. She was a non-smoker, with minimal alcohol intake and no illicit drug or over-the-counter medication use.

**Figure 1:**
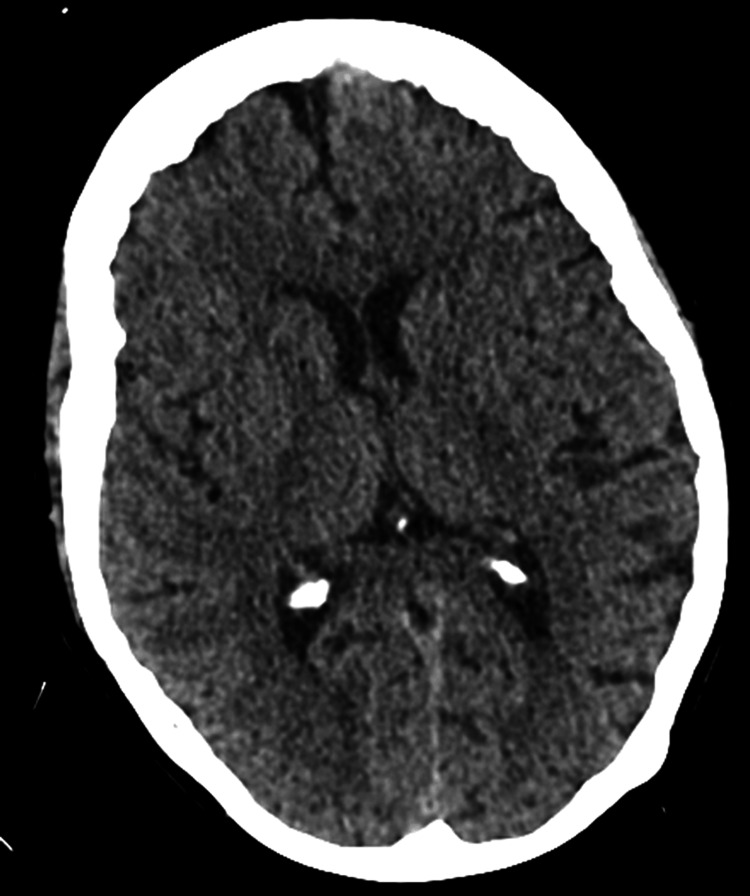
Patient's CT head scan demonstrating no acute abnormality with normal parenchymal volume and preserved grey-white matter differentiation CT: computed tomography

**Figure 2:**
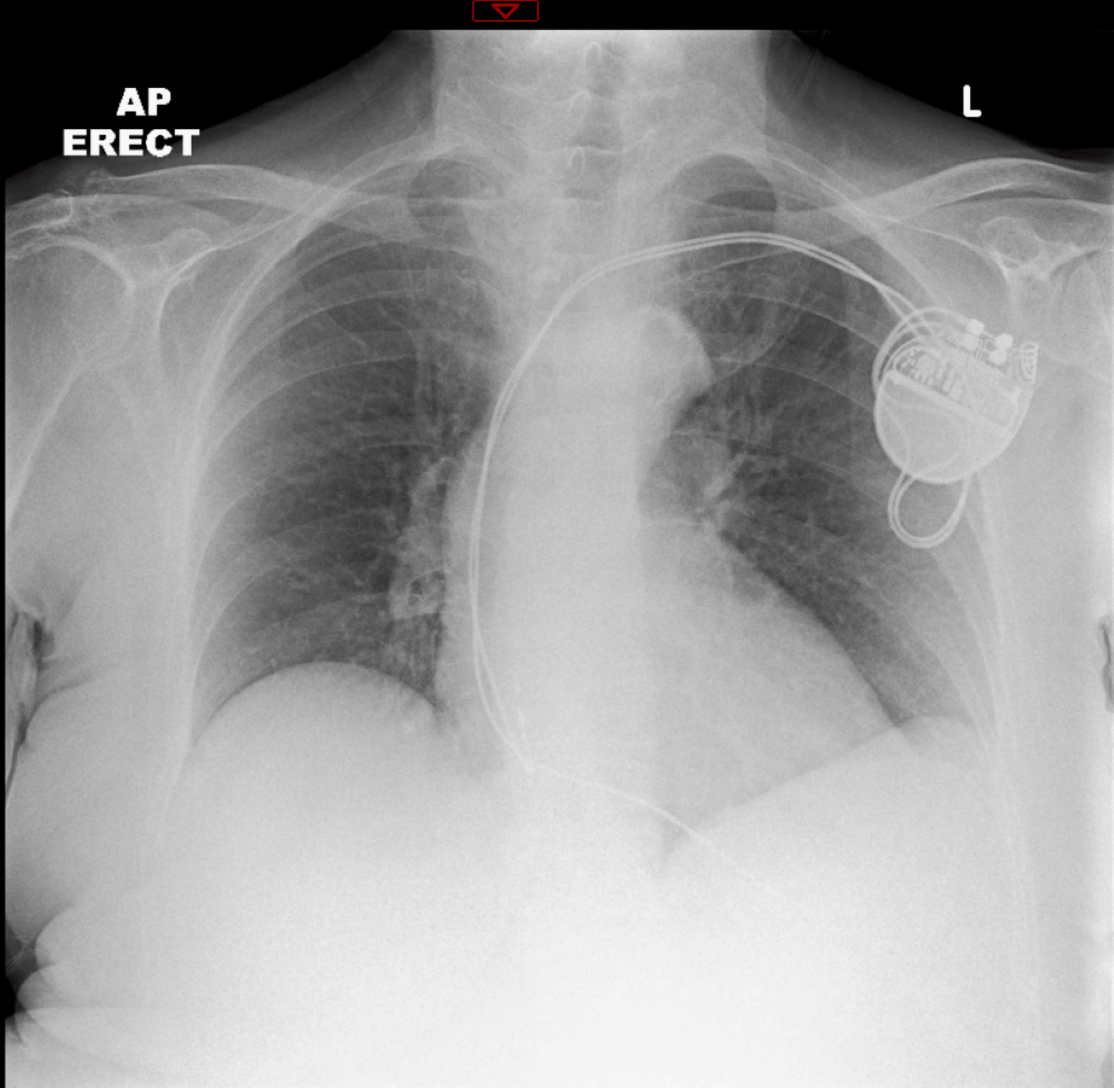
Patient's chest X-ray (anterior to posterior) demonstrating no acute abnormalities with a dual-chamber cardiac pacemaker in situ

**Figure 3:**
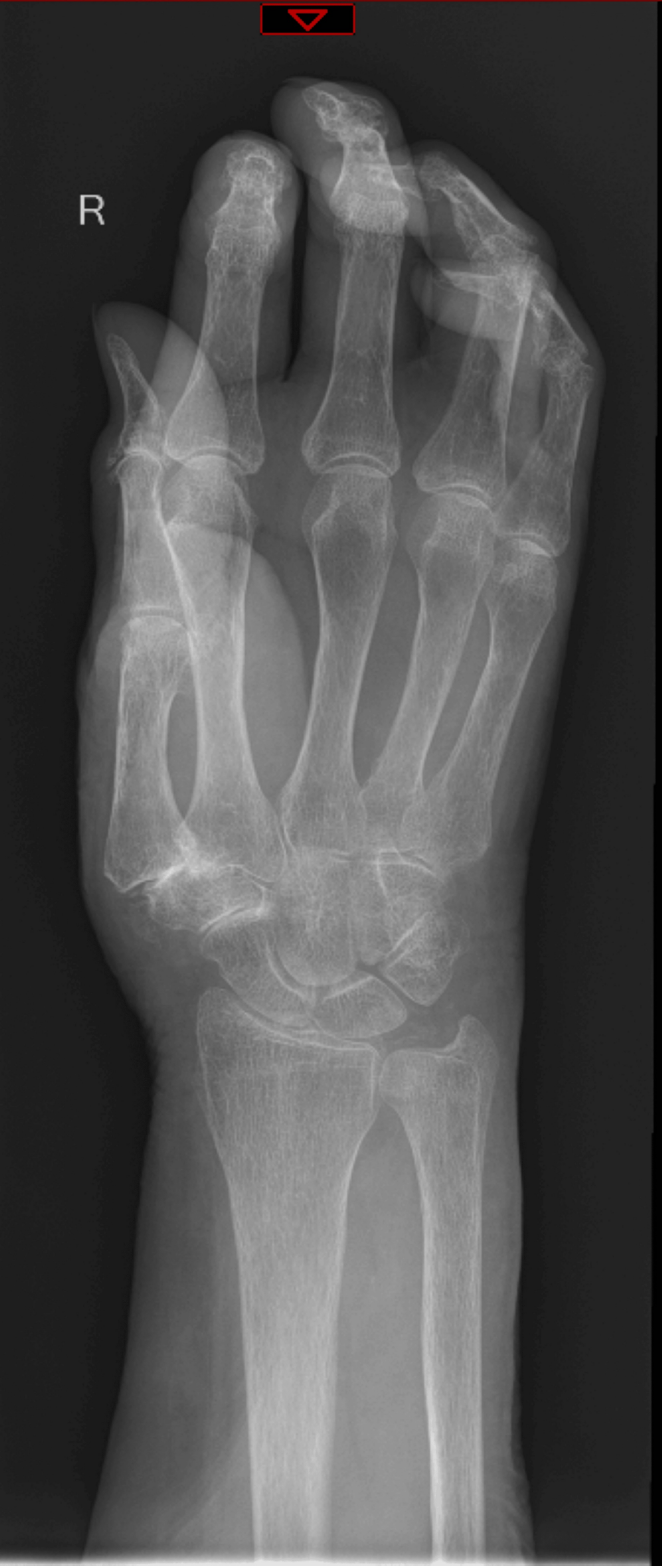
Patient's right hand X-ray and right wrist X-ray showing generalised osteopenia, severe degenerative change at the first carpometacarpal joint and several interphalangeal joints, with no plain film evidence of osteomyelitis

Admission bloods (Table 1) revealed mild neutrophilia, elevated C-reactive protein, anaemia, and mild chronic hyponatraemia (ranging from 132 to 134 mmol/L since 2012). Liver function tests, thyroid function tests, calcium, lactate, and vitamin B12 levels were within the reference range. Due to ongoing confusion, a computed tomography scan of the head (Figure 1) was performed, revealing no acute intracranial abnormalities with normal parenchymal volume and preserved grey-white matter differentiation. Her electrocardiogram showed a ventricular paced rhythm at 119 bpm, and a pacemaker check was satisfactory. Her chest X-ray (Figure 2) showed no acute abnormalities. Right wrist and hand x-rays (Figure 3) indicated generalised osteopenia, severe degenerative change at the first carpometacarpal joint and several interphalangeal joints, with no plain film evidence of osteomyelitis. Blood cultures and urine cultures revealed no growth.

**Table 1:** Patient's admission bloods with reference ranges. Abnormal values include a mild neutrophilia, mildly elevated C-reactive protein, anaemia, and a mild chronic hyponatraemia

Parameters	Patient values	Reference range
Haemoglobin	114	115-165 g/L
White blood cell count	10.1	4.0-11.0 x 10^9^/L
Neutrophil cell count	7.62	2.0-7.5 x 10^9^/L
C-reactive protein	46	0-5.0 mg/L
Sodium	130	133-146 mmol/L
Potassium	4.4	3.6-5.0 mmol/L
Adjusted calcium	2.45	2.2-2.6 mmol/L
Creatinine	49	50-98 umol/L
Urea	3.7	2.5-6.6 mmol/L
Estimated glomerular filtration rate	>60	>60 ml/min/1.73^2^
Alanine transaminase	16	10-50 U/L
Alkaline phosphatase	78	40-125 U/L
Bilirubin	16	3-21 umol/L
Thyroid-stimulating hormone	1.3	0.23-5.6 mU/L
Free thyroxine	16	9-28 pmol/L
Vitamin B12	324	180-2000 ng/L
Lactate	1.7	0.6-2.4 mmol/L

She was admitted to plastic surgery as per the local hospital’s protocol, which stipulates that patients presenting with cellulitis involving the hand should be admitted under the care of the plastic surgeons due to being a regional hand unit. She was commenced on intravenous vancomycin due to a documented flucloxacillin allergy, with clarithromycin switched to intravenous form as per local hospital protocol. After 24 hours, she was switched to a single oral agent: clarithromycin 500 mg twice daily. In the hospital, her suspected cellulitis improved, and her blood normalised with a significant reduction in C-reactive protein (13 mg/L).

**Figure 4:**
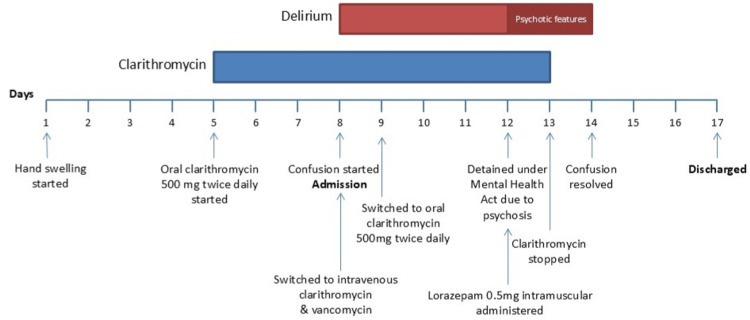
Course of admission. Days are numbered horizontally along the x-axis with key events noted. Duration of clarithromycin administration shown by the blue bar. Duration of delirium and psychosis shown by the red bar

Her care was transferred to geriatric medicine due to persistent confusion on day 3 with a 4AT score of 7 [7]. On day four of admission, her confusion and agitation worsened (4AT > 8), and she presented as suspicious (unwilling to answer questions) and distressed (entering other patients’ rooms and banging on the windows). She described delusional beliefs that nursing staff had been responsible for murder and persecutory beliefs that the nursing staff were conspiring against her. She had no apparent auditory or visual hallucinations. Subsequently, she made attempts to leave the hospital, resulting in an emergency detention under the Mental Health (Care and Treatment) (Scotland) Act 2003 [9]. Intramuscular (IM) lorazepam (0.5 mg) was administered, which helped reduce her distress. She remained confused but no longer a risk to herself and others; therefore, no further sedative medications were given. Clarithromycin was stopped due to the acknowledged risk of clarithromycin-induced psychosis [1]. Within 24 hours, she made a complete recovery and was oriented to time and place with no evidence of distress, unusual beliefs, or perceptual disturbances. She was discharged three days later. An outpatient physiotherapy entry from 16 days post-discharge details that the patient was able to provide a summary of her admission and was engaging well. Figure 4 summarises the admission course and the other causes of delirium considered and addressed throughout her admission. These included infection, pain, electrolyte disturbance, cardiac ischaemia, arrhythmia, alcohol excess/withdrawal, medications, hydration, constipation, and urinary retention [6].

## Discussion

Delirium is defined as a syndrome that encompasses an acute change in the patient’s attention, cognition, or awareness [6]. Wilson et al. suggested the prevalence of delirium in general medical inpatients is one in four [6]. Symptoms range from confusion to agitation, and in this patient, various drivers of delirium, previously noted in Figure 4, were considered and will be discussed [6].

It was postulated that the infection could have driven the neuropsychiatric disturbances [6]. However, blood cultures revealed no growth, and urine cultures obtained on days 5 and 6 of admission were negative for bacterial growth. Her chest x-ray also did not reveal signs of consolidation to suggest pneumonia. Upon examination, she presented with mild swelling and erythema on the dorsum of her hand, with no evidence of flexor sheath infection. She was systemically well with no fever. The cellulitis clinically resolved within two days of admission, but her altered mental state deteriorated with continued antibiotic administration, suggesting that infection was not the driver of her psychosis.

Systematic reviews have demonstrated an association between hyponatraemia and increased risk of delirium and cognitive impairment [10]. This patient’s hyponatraemia was chronic (132-134 mmol/L since 2012) and mild at 130 mmol/L. It normalised after intravenous fluids, but the neuropsychiatric disturbance persisted despite normal sodium levels.

Anaemia of chronic disease can increase delirium risk and may have increased her vulnerability to neuropsychiatric disturbances [11]. Her mild anaemia existed prior to admission and was just below normal reference ranges; therefore, it felt unlikely to be a significant contributor to her psychotic symptoms.

A diagnostic pacemaker check was satisfactory, excluding arrhythmias as a potential driver of delirium. Opioids are a recognised driver of delirium; therefore, the acute prescription of dihydrocodeine (30 mg four times daily) started by the plastics department was discontinued [6]. The single dose of IM lorazepam did help to reduce her distress, enabled nursing staff to attend to her personal care, and reduced the risk of physical harm to herself and others. However, neither stopping the opioid nor administering the lorazepam improved her psychotic or cognitive symptoms, and she remained confused [6]. Haloperidol was not prescribed due to concerns surrounding the lengthening of the corrected QT interval in combination with clarithromycin [12].

Cognitive impairment and frailty are known risk factors for delirium, with higher prevalences (>31%) in frail hospitalised patients [6]. However, this patient was neither frail, with a Clinical Frailty Score of 2, nor cognitively impaired [8]. Calming and reorientation strategies and pharmacological intervention were unsuccessful in managing this patient’s altered mental state [6]. The prominence of the persecutory delusions, which continued to deteriorate, necessitated the use of the Mental Health (Care and Treatment) (Scotland) Act 2003 [9]. This prompted consideration of alternative diagnoses, including the less common, but recognised, clarithromycin-induced psychosis [1,13]. After stopping clarithromycin, the psychotic symptoms rapidly ceased within 24 hours, with no evidence of agitation, distress, unusual beliefs or perceptual disturbance. The rapid resolution supported the hypothesis of clarithromycin-induced psychosis.

The Diagnostic and Statistical Manual of Mental Disorders (DSM-5) defines medication-induced psychosis as delusions, hallucinations and disorganised thinking/behaviour after drug consumption [3]. A recent study in 2020 [14] investigated 23 different antibiotics and demonstrated that clarithromycin had the highest odds ratio (9.48) for an adverse reaction of psychosis. Other risk factors may include renal impairment, prior psychiatric comorbidities, or older age, with an average age of 62 years [2,4]. Although the consensus indicates all age groups can be affected [2,4]. The aetiology of clarithromycin-induced psychosis is poorly understood [2,4]. Clarithromycin is metabolised in the liver by cytochrome P450, an isoenzyme from the CYP3A4 family responsible for the metabolism of multiple drugs [4,14]. Due to clarithromycin’s potent inhibition of CYP3A4, drug interactions could increase other medication concentrations, consequently inducing neuropsychiatric disturbances [4,14,15]. However, Table 2 demonstrates no interactions between the patient's medications and clarithromycin using the BNF interaction checker [12]. Additionally, CYP3A4 inhibition has been postulated to increase cortisol and prostaglandin levels. Several studies cite raised cortisol levels in psychotic episodes via increased hyperactivity of the hypothalamic-pituitary-adrenal axis [15,16]. Studies [2,4,15] also suggested clarithromycin metabolism alters neurotransmitters such as glutamate and gamma-aminobutyric acid (GABA). Another pathway stipulates that clarithromycin causes GABA-A antagonism, which can exert direct toxicity on the central nervous system (CNS) via excitatory effects, inducing psychosis [5,15]. Finally, a metabolite of clarithromycin (14-hydroxyclarithromycin) also exhibits CNS neurotoxic effects [2,15]. However, further research is required to investigate the mechanism(s) responsible [2].

**Table 2:** Patient’s medications, dosages, frequency and any interaction with clarithromycin according to the BNF interaction checker. No interactions were discovered BNF: British National Formulary

Medication	Dosage	Frequency	Interaction with clarithromycin
Paracetamol	1 g	As required (max four times daily)	No
Aspirin	75 mg	Once daily	No
Omeprazole	10 mg	Once daily	No
Candesartan	2 mg	Once daily	No

Clarithromycin-induced psychosis manifests within 10 days of commencing clarithromycin with total dosages of 1000 mg daily [2,4]. When clarithromycin is stopped, the neuropsychiatric symptoms of patients resolve promptly, as in this case [2,4]. One case report demonstrated that after ciprofloxacin was mistakenly prescribed for *Helicobacter pylori* treatment for one week and swapped for the correct clarithromycin, the patient exhibited neuropsychiatric symptoms after two days [2].

Limitations

The 4AT was not formally calculated after day 3 of admission. The 4AT was therefore retrospectively calculated based on the patient’s clinical notes, according to documentation of the Abbreviated Mental Test 4 and attention (“agitated”). This is reflected by a value of >8 in the case presentation.

## Conclusions

This case report supports mounting evidence that clarithromycin can cause a profound and rapid onset of psychosis. Delirium management is a common practice in geriatric medicine. This patient had no prior psychiatric comorbidities or cognitive concerns and presented with an unusual delirium, which persisted despite exclusion of alternative causes. By recognising this rare but known adverse effect of clarithromycin, the patient’s psychotic symptoms rapidly resolved within 24 hours. This reinforces the need for awareness of rare neuropsychiatric reactions to clarithromycin, to recognise this relatively unusual presentation, and, more importantly, any future unusual delirium presentations that fail to respond to conventional delirium management.
